# *Placobdelloides sirikanchanae* sp. nov., a new species of glossiphoniid leech and a parasite of turtles from lower southern Thailand (Hirudinea, Rhynchobdellida)

**DOI:** 10.3897/zookeys.882.35229

**Published:** 2019-10-23

**Authors:** Poramad Trivalairat, Krittiya Chiangkul, Watchariya Purivirojkul

**Affiliations:** 1 Animal Systematics and Ecology Speciality Research Unit, Department of Zoology, Faculty of Science, Kasetsart University, 50 Ngam Wong Wan Road, Chatuchak, Bangkok, 10900, Thailand Kasetsart University Bangkok Thailand

**Keywords:** Clitellata, *
Cyclemys
*, Glossiphoniidae, Hirudinea, leaf turtle, Songkhla

## Abstract

**Abstract** A new species of glossiphoniid leech, *Placobdelloides sirikanchanae***sp. nov.**, is reported in the Asian leaf turtle (*Cyclemys dentata*) and the dark-bellied leaf turtle (*C. enigmatica*) from Songkhla Province, southern Thailand. The examination of morphological characters revealed that this new species is similar to *P. siamensis* (Oka, 1917), a common turtle leech species found in Thailand. *Placobdelloides sirikanchanae***sp. nov.** demonstrates distinct morphological characters, with an elongated, narrow body, 13–17 well-developed knob papillae on each annulus, dark brown to greenish dorsal color with a crimson median line, the absence of a scarlet dot, different male and female gonopore distributions, a rough posterior sucker with a random pit distribution, and 104–115 eggs per clutch. The phylogenetic relationships of COI-ND1 genes were clarified and shown to be distinct from those of *P. siamensis*. Additionally, habitat preferences tended toward low oxygen conditions such as puddles or water patches on rubber plantations.

## Introduction

Glossiphoniid leeches are characterized as the only annelids that have parental care behavior by carrying cocoons and juveniles directly on the ventral surface for protection and feeding (Sawyer 1986; Siddall et al. 2005). *Placobdelloides* Sawyer, 1986 is a genus of jawless leech species in the most diverse family Glossiphoniidae, which are distributed in freshwater habitats on all continents except Antarctica (Grube 1866; Benham 1907; Johansson 1909; Harding 1920, 1924; Oka 1925; Ingram 1957; Baugh 1960; Cott 1961; Soós 1969; Mason 1974; Chandra 1977; Oosthuizen 1979; Govedich et al. 2002; Nesemann et al. 2004; McKenna et al. 2005). This genus has a protrusible proboscis for both blood-feeding and tissue meals on vertebrates (Soós 1969; Sawyer 1986; Govedich 2001; Govedich et al. 2002; Tucker et al. 2005). Glossiphoniid leeches can be used as alkalinity stress indicators of their ecosystems and they are also vectors of apicomplexan blood parasites of aquatic vertebrates and are therefore very important in both ecology and the environment (Grantham and Hann 1994; Siddall and Burreson 1994).

*Placobdelloides siamensis* (Oka, 1917) is the only leech species currently reported from several different turtles of the family Geoemydidae in Thailand, which commonly inhabit flowing-water ecosystems (Brophy 2004; Das 2010; Chiangkul et al. 2018): the Southeast Asian box turtle, *Cuora amboinensis* Daudin, 1802; yellow-headed temple turtle, *Heosemys annandalii* (Boulenger, 1903); Malayan snail-eating turtle, *Malayemys macrocephala* (Gray, 1859); Mekong snail-eating turtle, *M. subtrijuga* (Schlegel & Müller, 1845); Khorat snail-eating turtle, *M. khoratensis* Ihlow et al., 2016; and the black marsh turtle, *Siebenrockiella crassicollis* (Gray, 1831). In this study, *Placobdelloides sirikanchanae* sp. nov. is described as the second member of the genus found on the turtle species in the family Geoemydidae, from nonflowing water habitats in Songkhla Province, southern Thailand.

This study presents the first report of the use of a combination of morphological and molecular techniques to describe a new leech species that parasitizes Asian leaf turtles, *Cyclemys dentata* (Gray, 1831) and dark-bellied leaf turtles, *C. enigmatica* Fritz et al., 2008. This newly discovered turtle leech is here presented along with new information about its identification and geographic distribution in Thailand.

## Materials and methods

### Leech collection and preservation

Leech specimens were collected from two different turtle species at six different collecting sites. Seven leaf turtles (three individuals of *C. dentata* and four individuals of *C. enigmatica*) were collected from the bottom of small muddy puddles or patches of approximately 20–30 cm depth in rubber plantations in Sadao District, Songkhla Province (6°62'57.7"N, 100°41'12.7"E) on 21 October 2018. Leeches were removed from the body and shell of each turtle using forceps and then stored in sealed bottles with water from the capture sites to keep them alive. The carapace length was measured for all turtles, after which they were released back into their capture sites when finished.

Leeches were maintained in a glass container (10×12×8 cm^3^) half full of puddle water and fitted with an oxygen-pumping machine for behavioral study in the laboratory. Afterward, some individuals were preserved in absolute ethanol in a relaxed stage for scanning electron microscopy (SEM) and molecular techniques, while still others were preserved in 70% ethanol in a relaxed stage for identification.

### Morphological study

Each specimen was examined for eye number and placement, annulation, digestive system (including the number and structure of gastric ceca), and reproductive system, following Sawyer (1986) under an MVX10 Research Macro Zoom microscope (Olympus) at 250× magnification. For scanning electron microscopy (SEM), leeches were preserved in absolute alcohol, dried using the critical point drying technique (CPD), and coated in gold, and their morphology was studied using a Quanta 450 Scanning Electron Microscope equipped with an Oxford Instrument X-Max (Kruger and Du Preez 2015).

### Molecular analysis

The leech specimens in absolute ethanol were sectioned into two equal pieces. The posterior part was used for DNA extraction with TIANamp Genomic DNA Kit (catalog number DP304-02; TIANGEN Biotech (Beijing) Co., Ltd., Beijing) while the anterior part was stored in absolute ethanol to be used later for a DNA sample stock. For the proteinase K treatment step, tissue samples were lysed for two hours at 58°C. The DNA was eluted from the spin column with 200 µl of buffer.

Polymerase chain reactions (PCR) were prepared using the EP0402 TAQ DNA POLYMERASE. Two mitochondrial gene fragments were amplified namely, cytochrome *c* oxidase subunit I (CO-I) and nicotinamide adenine dinucleotide dehydrogenase subunit I (ND-1) following Light and Siddall (1999). The CO-I universal primers used were: LCO1490 (5’-GGTCAACAAATCATAAAGATATTGG-3’) and HCO2198 (5’-TAAACTTCAGGGTGACCAAAAAATCA-3’) (Folmer et al. 1994). The ND-I primers used were LND300 (5’-TGGCAGAGTAGTGCATTAGG-3’) and HND1932 (5’-CCTCAGCAAAATCAAATGG-3’) (Light and Siddall 1999). Final volumes of PCR reactions were 30 µl with 3 µl of leech genomic DNA added per reaction. DNA was amplified under the following PCR conditions: 94 °C for 5 min; 35 cycles of 94 °C for 30 sec, 50 °C for 30 sec, and 72 °C for 45 sec; 72 °C for 7 min. PCR products were purified and sequenced by Macrogen Korea. The sequences obtained were than submitted to GenBank (Table 1).

**Table 1.** GenBank accession numbers for leech sequences used in the phylogenetic analysis of *Placobdelloides*.

Taxon	Locality	GenBank accession numbers	
		COI	ND1
Ingroup			
*Placobdelloides sirikanchanae* sp. nov.	Songkhla, Thailand	MK282428	MK282433
	Songkhla, Thailand	MK282429	MK282434
	Songkhla, Thailand	MK282430	MK282435
	Songkhla, Thailand	MK282431	MK282436
	Songkhla, Thailand	MK282432	MK282437
*Placobdelloides jaegerskioeldi* (Johansson, 1909)	Sudan, South Africa	AY962463	AY962450
*Placobdelloides multistriatus* (Johansson, 1909)	Louisiana, USA	DQ414338	DQ414383
*Placobdelloides siamensis* (Oka, 1917)	Bangkok, Thailand	AY962449	AY962462
	Bangkok, Thailand	MH777415	MH777409
	Bangkok, Thailand	MH777416	MH777410
	Bangkok, Thailand	MH777417	MH777411
	Bangkok, Thailand	MH777418	MH777412
	Bangkok, Thailand	MH777419	MH777413
	Bangkok, Thailand	MH777420	MH777414
	Udon Thani, Thailand	MN221458	MN242784
	Udon Thani, Thailand	MN221459	MN242785
	Udon Thani, Thailand	MN221460	MN242786
Outgroup			
*Alboglossiphonia heteroclita* (Linnaeus, 1761)	Michigan, USA	AF116016	AY047339
*Alboglossiphonia quadrata* (Moore, 1949) Sawyer, 1986	Namibia, South Africa	AY962455	AY962441
*Alboglossiphonia weberi* (Blanchard, 1897b)	Hawaii, USA	AY962453	AY962440
*Batracobdelloides tricarinata* (Blanchard, 1897a)	Hoedspruit, South Africa	AY962457	AY962445
*Glossiphonia baicalensis* (Stchegolew, 1922)	Lake Baikal, Russia	AY047329	AY047355
*Glossiphonia complanata* (Linnaeus, 1758)	United Kingdom	MF458715	AY047345
*Glossiphonia concolor* (Apathy, 1888)	Kila River, Sweden	AY962458	AY962446
*Glossiphonia elegans* (Verrill, 1872)	Connecticut, USA	AY047322	AY047335
*Glossiphonia verrucata* (Müller, 1844)	Rio s’ Adde, Italy	AY962459	AY962447
*Helobdella fusca* (Castle, 1900)	Michigan, USA	AF329038	AF329061
*Helobdella robusta* (Shankland, Bissen & Weisblat, 1992)	Sacramento River, USA	MF067148	MF067201
*Hemiclepsis marginata* (Müller, 1774)	Étang de la Musse, France	AF003259	AY047336
*Hirudo medicinalis* (Linnaeus, 1758)	Gotland, Sweden	HQ333517	KU672396
*Marsupiobdella africana* Goddard & Malan, 1912	South Africa	AF116015	AY047347
*Placobdella montifera* (Moore, 1906)	Washington, USA	MF067129	MF067212
*Placobdella pediculata* (Hemingway, 1908)	Lake Pepin, USA	MF067121	MF067222
*Theromyzon bifarium* Oosthuizen & Davies, 1993	North USA	AY047330	AY047356
*Theromyzon tessulatum* (Müller, 1774)	Europe	AY047318	AY047338

### Statistical analysis

The DNA sequences were aligned using ClustalW v. 1.83 (Thompson et al. 1994) and analyzed using MEGA6 v. 6 (Tamura et al. 2013) for maximum likelihood analysis and MrBayes v. 3.1.2 (Ronquist and Huelsenbeck 2003) for Bayesian analysis.

The maximum likelihood analysis consisted of 2000 tree search replicates, with 25 initial GAMMA rate categories and final optimization using four GAMMA shape categories. Bootstrap values were calculated using 2000 pseudoreplicates of the rapid bootstrap algorithm. Bayesian analysis was run for 20 million generations with trees sampled every 100 generations with a general time reversible (GTR) model and GAMMA distribution of nucleotide rates for all partitions. Burn-in was set to 10%. Bootstrap values ≥70% for maximum likelihood analysis and Bayesian posterior probabilities of ≥95% were considered a priori as being indicators of highly supported nodes (Felsenstein 2004).

## Results

### Turtle body size and prevalence

**Figure 1.**
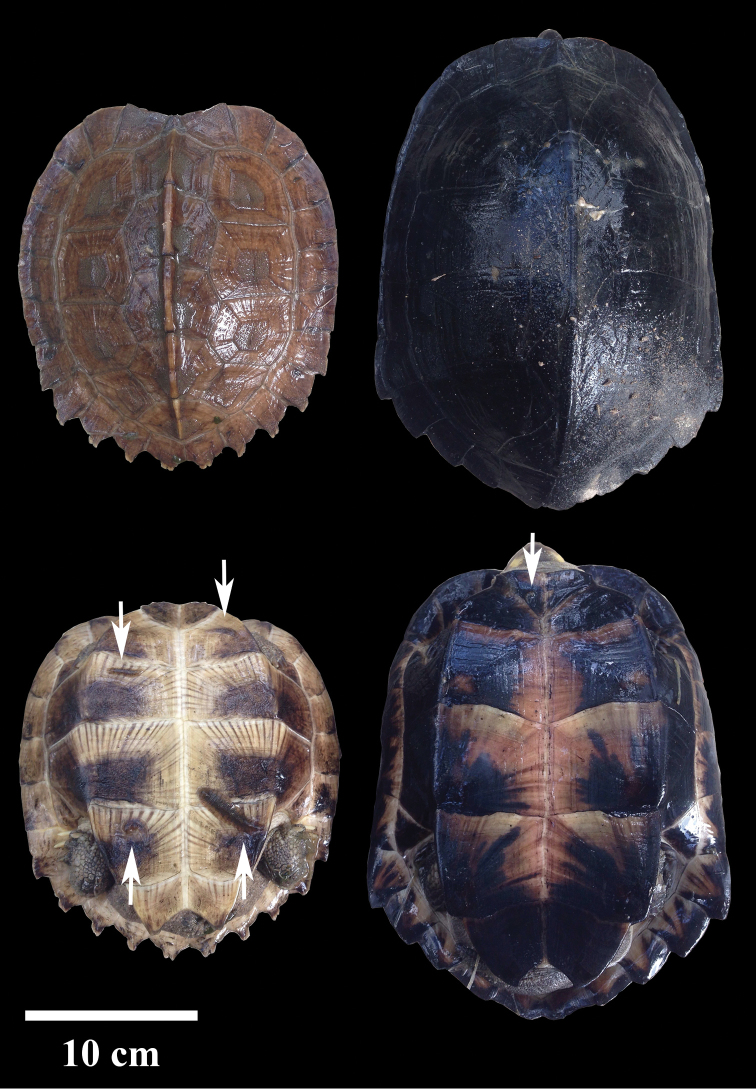
Live *Placobdelloides sirikanchanae* sp. nov. (arrows) on the Asian leaf turtle (*Cyclemys dentata* (Gray, 1831)) (left) and the dark-bellied leaf turtle (*C. enigmatica* Fritz et al., 2008) (right): carapace (lower), plastron (upper).

In total, six muddy puddles on rubber plantations (6°62'57.7"N, 100°41'12.7"E) were inhabited by two turtle species: *Cyclemys dentata* and *C. enigmatica* (Figure 1). Three individuals of *C. dentata* had a mean carapace length of 19.20 ± 2.36 cm (min-max: 17.50–21.90 cm), and four individuals of *C. enigmatica* had a mean carapace length of 24.02 ± 0.66 cm (min–max: 22.7–26.3 cm). Each leaf turtle had 2–3 individuals of *Placobdelloides sirikanchanae* sp. nov. attached to it. In total, twenty individuals of *P. sirikanchanae* were removed, mostly from the carapace or plastron surfaces. The turtles at the collecting sites were seen to be predating on small fishes and *Rhacophorus* tadpoles.

### Species description

#### Placobdelloides

Taxon classificationAnimaliaRhynchobdellidaGlossiphoniidae

Sawyer, 1986

A3220BF3-D88C-577F-99EA-6CE703104F1D

##### Type species.

*Placobdelloides multistriata* (Johansson, 1909) by original designation.

##### Genus diagnosis.

eyes one pair, esophageal organ, crop caeca seven pairs, mouth pore terminal (Oosthuizen 1979).

##### Genus distribution.

*Placobdelloides* species can be found in Africa (*P. fimbriata* (Johansson, 1909); *P. jaegerskioeldi* (Johansson, 1909); *P. multistriata* (Johansson, 1909)), Australia and United States, eastward to India (*P. fulva* (Harding, 1924); *P. emydae* (Harding, 1920); *P. undulata* (Harding, 1924); *P. horai* (Baugh, 1960); *P. indica* (Baugh, 1960)), Southeast Asia (*P. siamensis* in China and Thailand; *P. okadai* (Oka, 1925) in China; *P. okai* (Soós, 1969); *P. stellapapillosa*Govedich et al., 2002 in Malaysia and Singapore), and throughout Australia and New Zealand (*P. octostriata* (Grube, 1866); *P. maorica* (Benham, 1907); *P. bancrofti* (Best, 1931); *P. bdellae* (Ingram, 1957)).

#### Placobdelloides sirikanchanae sp. nov.

Taxon classificationAnimaliaRhynchobdellidaGlossiphoniidae

0F491ADD-3720-524E-B87D-884F7A6D6600

http://zoobank.org/CE96B3D0-7E8F-47D5-8212-FCFFBFA907FF

Figures 2 , 3 , 4 , 5 , 6 , 7 , 8 , 9

##### Material examined.

***Holotype*** (ZMKU-ANN-0006), puddle on rubber plantation, Sadao District, Songkhla Province, Thailand (6°62'57.7"N, 100°41'12.7"E), 21 October 2018. ***Paratypes*** (nine individuals, ZMKU-ANN-0007 to 0015), same locality data as the holotype. All collected specimens were kept in 70% alcohol and deposited at the Zoological Museum of Kasetsart University (ZMKU), Department of Zoology, Faculty of Science, Kasetsart University (13°50'53.6"N, 100°33'47.3"E) on 23 November 2018.

##### Diagnosis.

This species can be recognized from its elongated, narrow body, crimson median dorsal line, rich dark green pigmentation, 13–17 well-developed knob papillae on each annulus, symmetrical dorsal papillae between the left and right body sides, male gonopore on XIa1/a2, female gonopore on XIa3/XIIa1, amorphous salivary glands, smooth surface with random pits inside the anterior sucker, and rugged surface with randomly distributed pits inside the posterior sucker.

##### Description of holotype.

**Figure 2.**
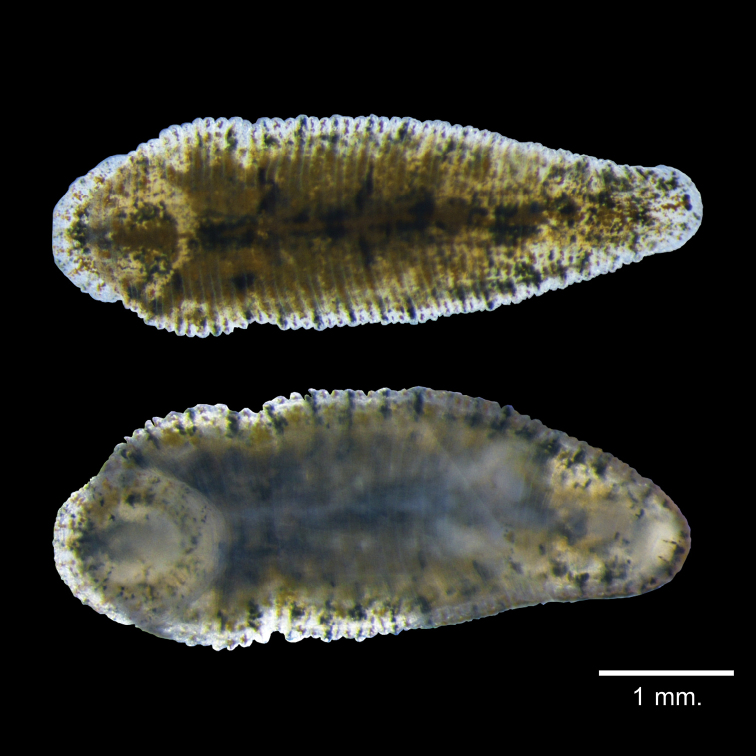
Dorsal surface (upper) and ventral surface (lower) of the live holotype of *Placobdelloides sirikanchanae* sp. nov.

**Figure 3.**
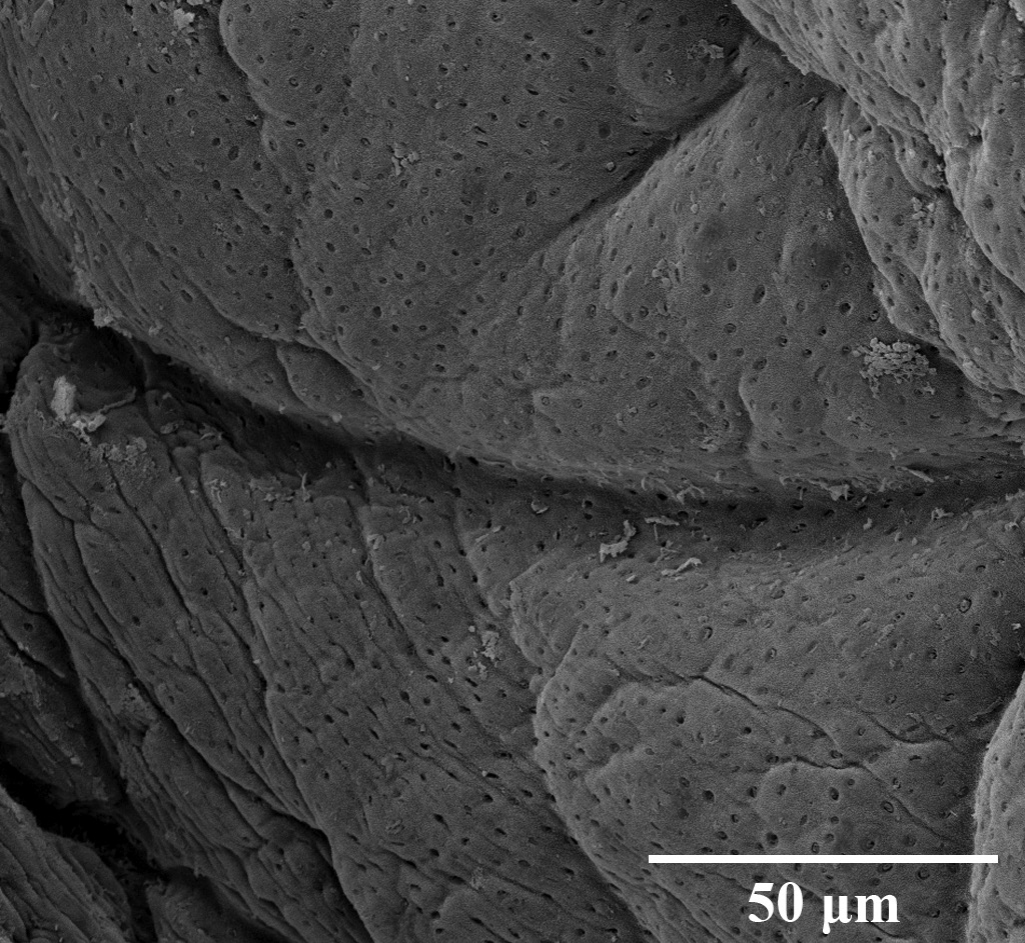
Scanning electron micrograph of anterior sucker of the paratype ZMKU-ANN-0009 of *Placobdelloides sirikanchanae* sp. nov. showing smooth surface with numerous pits.

**Figure 4.**
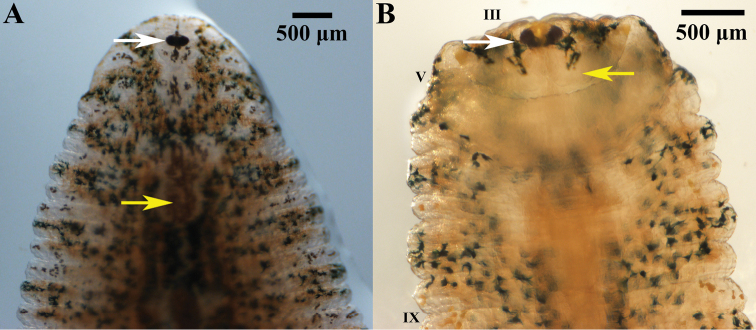
Anterior part of the live holotype of *Placobdelloides sirikanchanae* sp. nov. (**A**) Dorsal surface showing eyes touching on somite III (white arrow) and crimson red dorsal median line (yellow arrow), (**B**) Ventral surface showing rolled anterior lip (yellow arrow) and eyes (white arrow).

**Figure 5.**
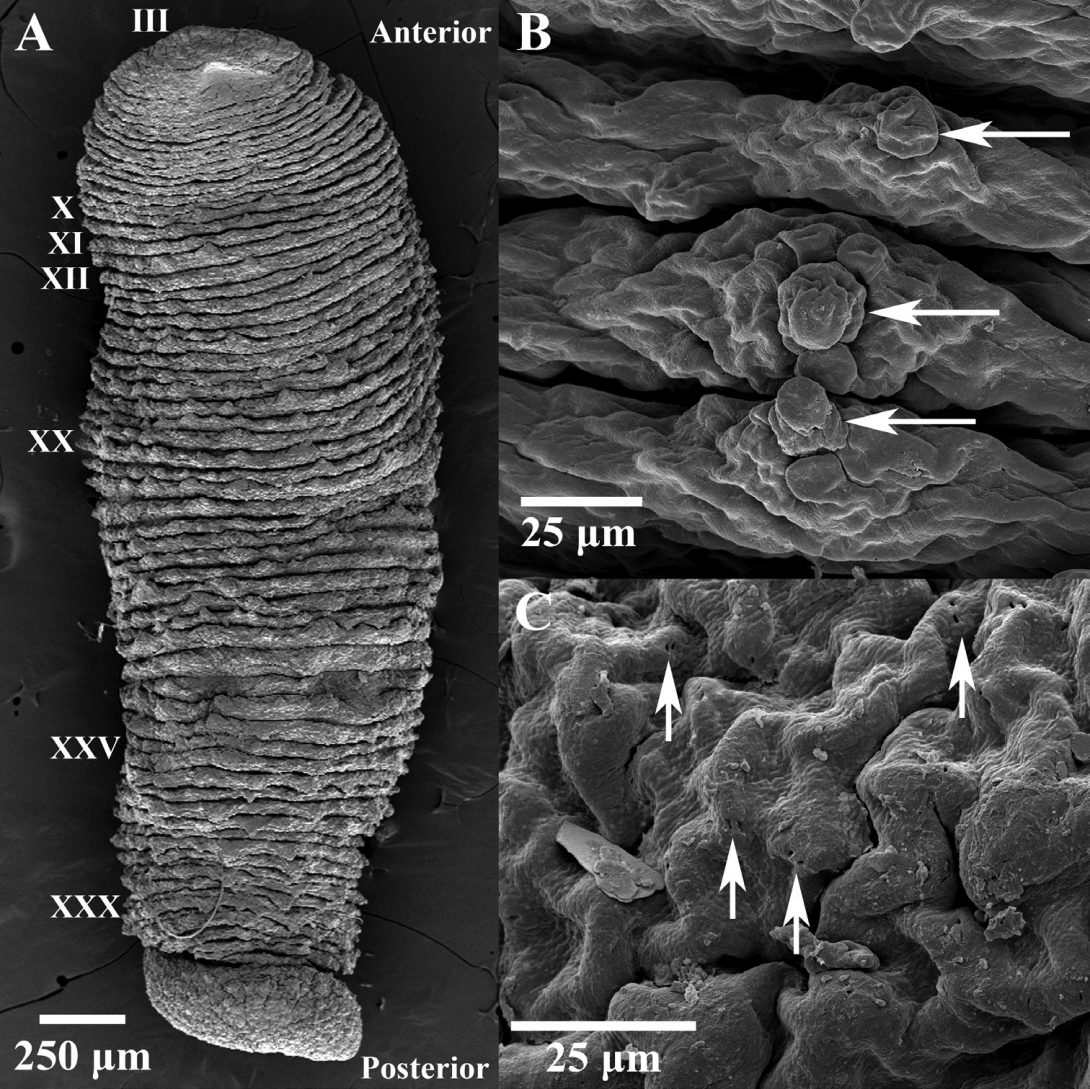
Scanning electron micrograph of dorsal surface of the paratype ZMKU-ANN-0010 of *Placobdelloides sirikanchanae* sp. nov. **A** Dorsal surface of the complete body **B** Dorsal papillae (arrows) **C** Respiratory pores on dorsal surface (arrows).

**Figure 6.**
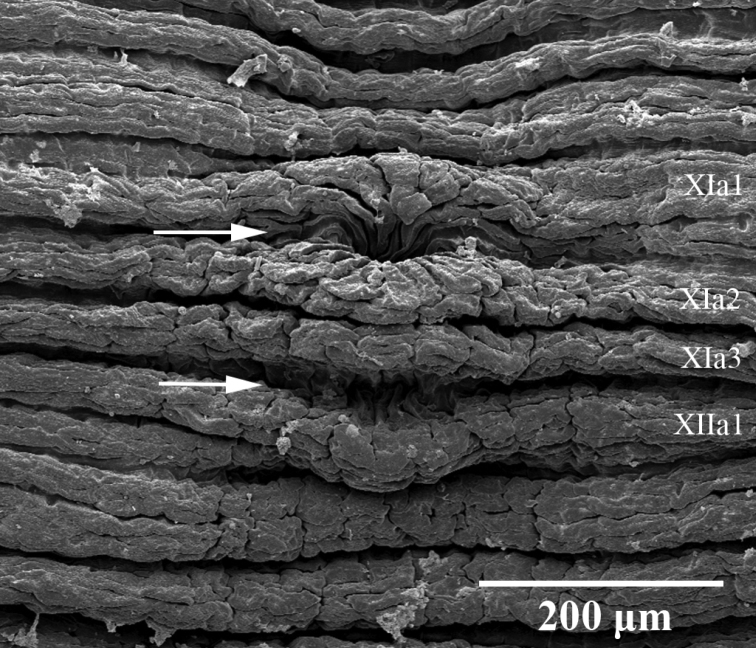
Scanning electron micrograph of ventral surface of the paratype ZMKU-ANN-0009 of *Placobdelloides sirikanchanae* sp. nov. showing gonopore arrangement. Upper arrow points to the male gonopore and lower arrow to the female gonopore.

**Figure 7.**
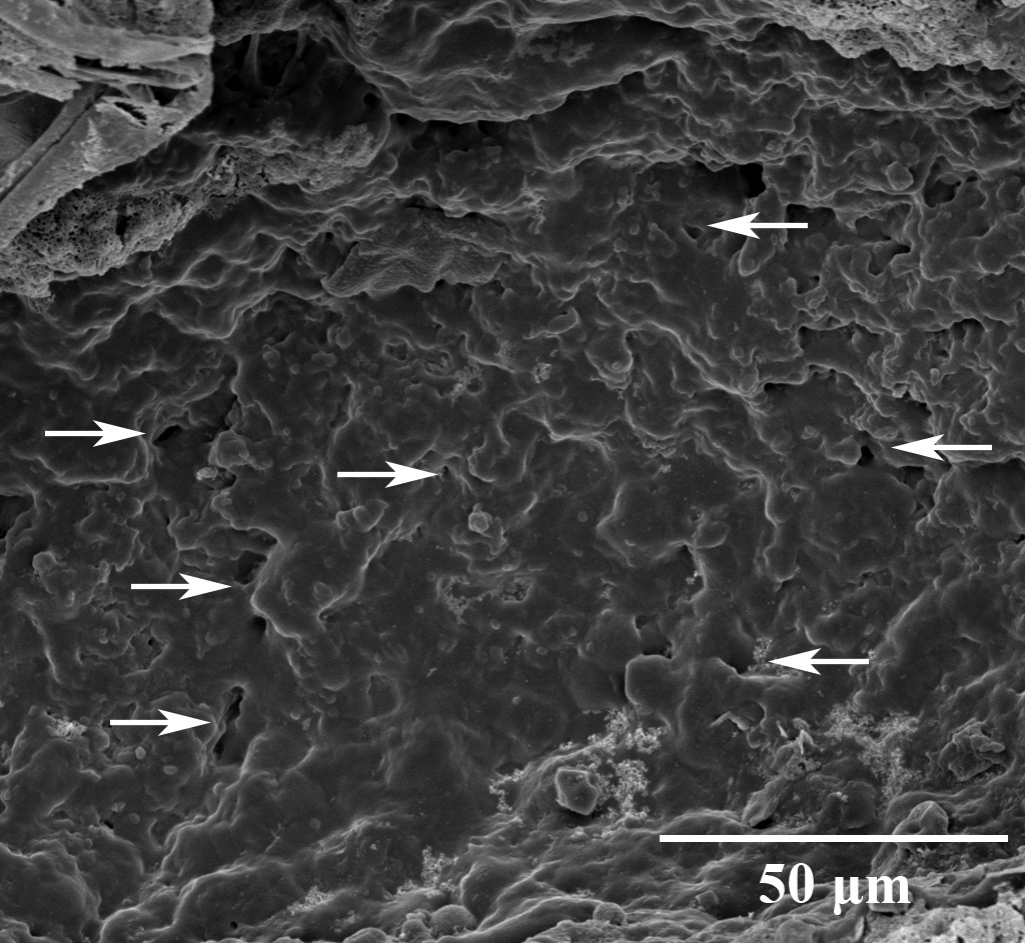
Scanning electron micrograph of posterior sucker of the paratype ZMUKU-ANN-0009 of *Placobdelloides sirikanchanae* sp. nov. showing rough surface with random pit distribution (white arrows).

***External morphology*.** A mature *Placobdelloides sirikanchanae* sp. nov. (ZMKU-ANN-0006) has an elongated, dorso-ventrally flattened, tri-annulate body (Figure 2). The relaxed body length from the anterior tip to the posterior sucker is 20.83 mm. The widest point of the relaxed body (annuli 35; XV) is 4.21 mm. The cup-shaped anterior sucker diameter is 1.17 mm. The anterior sucker surface is smooth with numerous pits distributed inside (Figure 3; paratype ZMKU-ANN-0009). One pair of dark spherical eyes touch each other on somite III (Figure 4). The entire dorsal surface is quite rough, with 13–17 well-developed knob papillae present on each annulus (Figure 5; paratype ZMKU-ANN-0010). The dorsal papillae present a symmetrical pattern between the left and right sides of the crimson median line. The dorsal color is dark brown to greenish. The numerous respiratory pores are randomly distributed on the dorsal surface. The ventral surface is transparent and smooth. Two gonopores are located around the neck region and separated by two annuli. The male gonopore is situated in a furrow of XIa1/a2, between annuli 23 and 24 (Figure 6; paratype ZMKU-ANN-0009). The female pore lies in a furrow of XIa3/XIIa1, between annuli 25 and 26. The anus opening is on the dorsal furrow anterior to the last annulus (69; XXXIV). The posterior sucker diameter is 2.08 mm. The posterior sucker surface is rough with randomly distributed pits inside (Figure 7; paratype ZMKU-ANN-0009).

***Annulation*.** Somites I-III are uni-annulate, IV and V are biannulate (annuli 4–7), VI-XIV are tri-annulate (annuli 8–34), XV-XVIII are uni-annulate (annuli 35–38), XIX-XXV are tri-annulate (annuli 39–59), XXVI is biannulate (annuli 60–61), and XXVII-XXXIV are uni-annulate (annuli 62–69).

##### Internal morphology.

***Digestive system***: A cylindrical slender proboscis resides in a membranous sheath that protrudes through the lip of the posterior subterminal mouth (Figure 9). The proboscis sheath line is on VIa1-Xa2 (annuli 8–21). Amorphous salivary glands are packed on Xa2-XIa3 (annuli 21–25), followed by the esophageal glands on XIa1-XIIa1 (annuli 23–26). Each esophageal gland has a salivary duct that joins it to each side of the esophagus. Seven pairs of crop cecae are on XIIIa2-XXIIIa1 (annuli 30–51) with the last pair on XXIIIa1-XXXI (annuli 51–66) being diverted and extended posteriorly into four post cecae. Four pairs of diverticulated intestine are on XXIIIa1-XXXIII (annuli 51–68). A simple narrow rectum resides on XXVIa2-XXXIV (annuli 61–69) and opens dorsally at the anus in a furrow anterior of the last somite (XXXIV, annulus 69).

***Reproductive system*.** The male gonopore rim is thick and curled. The ejaculatory bulb on XIa2-XIIa2 (annuli 24–27) is an apple-like sac opening into the vas deferens. Two vas deferens extend posteriorly and recurve in front of post ceca anteriorly to connect to the testisacs. Six pairs of ovoid testisacs are present, and each is located in the space between a pair of crop cecae. The female gonopore rim is thinner and smoother than that of the male. The spermatheca is a rectangular sac on XIIa2-XIIIa3 (annuli 27–31), which opens into bifurcated ovisacs.

##### Variation.

***External morphology*.** The average relaxed body length is 10.77 mm long (range 7.62–40.39 mm, *N* = 20), and the average relaxed body width at the widest point (annuli 35, XV) is 3.96 mm (range 3.52–4.89 mm, *N* = 20). The average anterior sucker diameter is 1.08 mm (range 0.93–1.42 mm, *N* = 20). The average posterior sucker diameter is 1.94 mm (range 1.70–2.60 mm, *N* = 20), half the size of the maximum body width.

**Figure 8.**
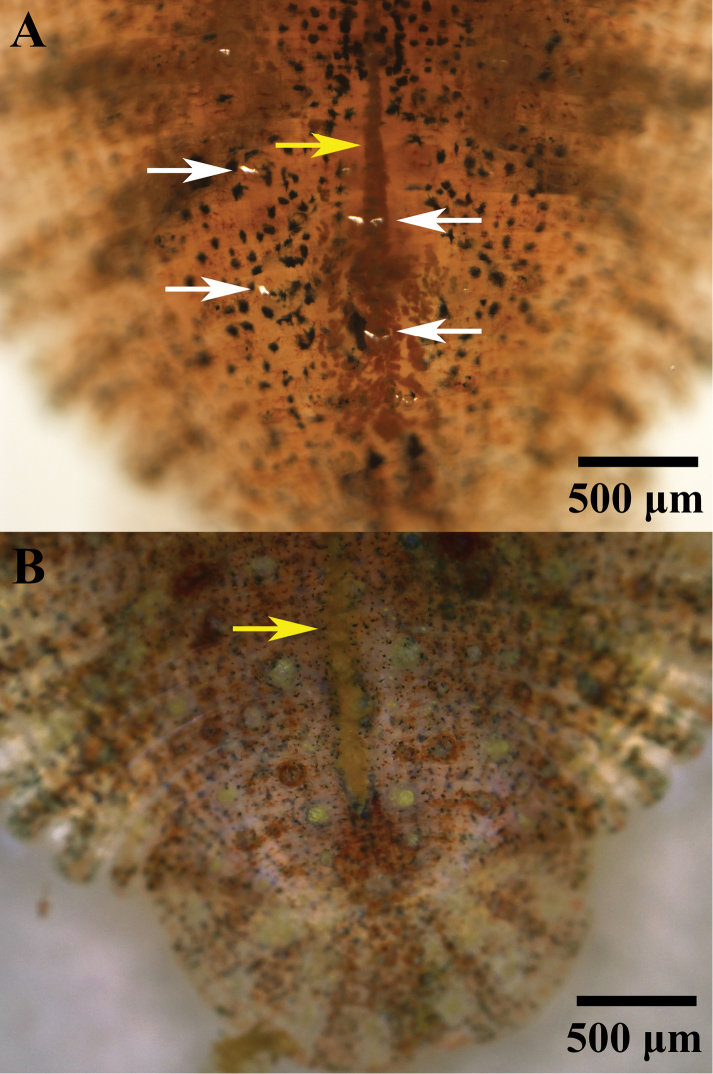
Posterior part of live specimens **A** holotype *Placobdelloides sirikanchanae* sp. nov. showing dorsal crimson median line (yellow arrow) with numerous scattered dark green pigments. Bubbles are emerging from the respiratory pores (white arrows) **B***Placobdelloides siamensis* (Oka, 1917) from Bangkok, Thailand showing yellow median line (yellow arrow) with numerous scattered yellow pigments.

***Color*** in life is uniformly dark brown to greenish, with randomly distributed dark brown, red, yellow, and especially rich dark green pigments. There is a crimson median line present dorsally from the neck region to the posterior sucker (Figure 8). On the margin of the body, brown, dark green and yellow spots are present along the posterior sucker. The ventral surface is transparent.

***Reproductive system*.** The length of the ovisacs depends on the reproductive stage. During the normal, non-reproductive period, ovisacs are present on XIIIa1-XIVa1 (annuli 29–32), but they can extend from XIIIa1 to XXa1 (annuli 29 to 42 (4^th^ pair of crop cecae)) during the gestational period.

##### Molecular description.

Molecular comparisons based on *p*-distances among five specimens of *P. sirikanchanae* sp. nov. from a rubber plantation in the Sadao District, Songkhla Province, Thailand revealed a difference of 2.5–6.2% for 518 nucleotides of COI (GenBank MK282428–MK282432) and 1.3–3.3% for 555 nucleotides of ND1 (GenBank MK282433–MK282437) (see Tables 2, 3). The five specimens of *P. sirikanchanae* revealed differences based on *p*-distances of 10.4–27.7% for the COI gene and 5.4–6.9% for ND1 compared to ten specimens of *P. siamensis* (GenBank AY962449, MH777415–MH777420, MN221458–MN221460 for COI, and AY962462, MH777409–MH777414, XX123456–XX13456 for ND1) collected from Bangkok and Udon Thani Province, Thailand; differences of 19.3–21.7% for the COI gene and 15.1–15.8% for ND1 compared to a specimens of *P. multistriatus* (GenBank DQ414338 for the COI gene, and DQ414383 for the ND1 gene) collected from Louisiana, USA; and differences of 21.0–23.5% for the COI gene and 15.1–16.0% for ND1 compared to a specimen of *P. jaegerskioeldi* (GenBank AY692463 for COI, and AY962450 for ND1) collected from Sudan, South Africa. The Bayesian inference and maximum-likelihood trees of the COI and ND1 genes of the glossiphoniid leeches indicated high posterior probabilities and bootstrap support values for divergence between *P. sirikanchanae* and *P. siamensis* (Figure 10).

**Figure 9.**
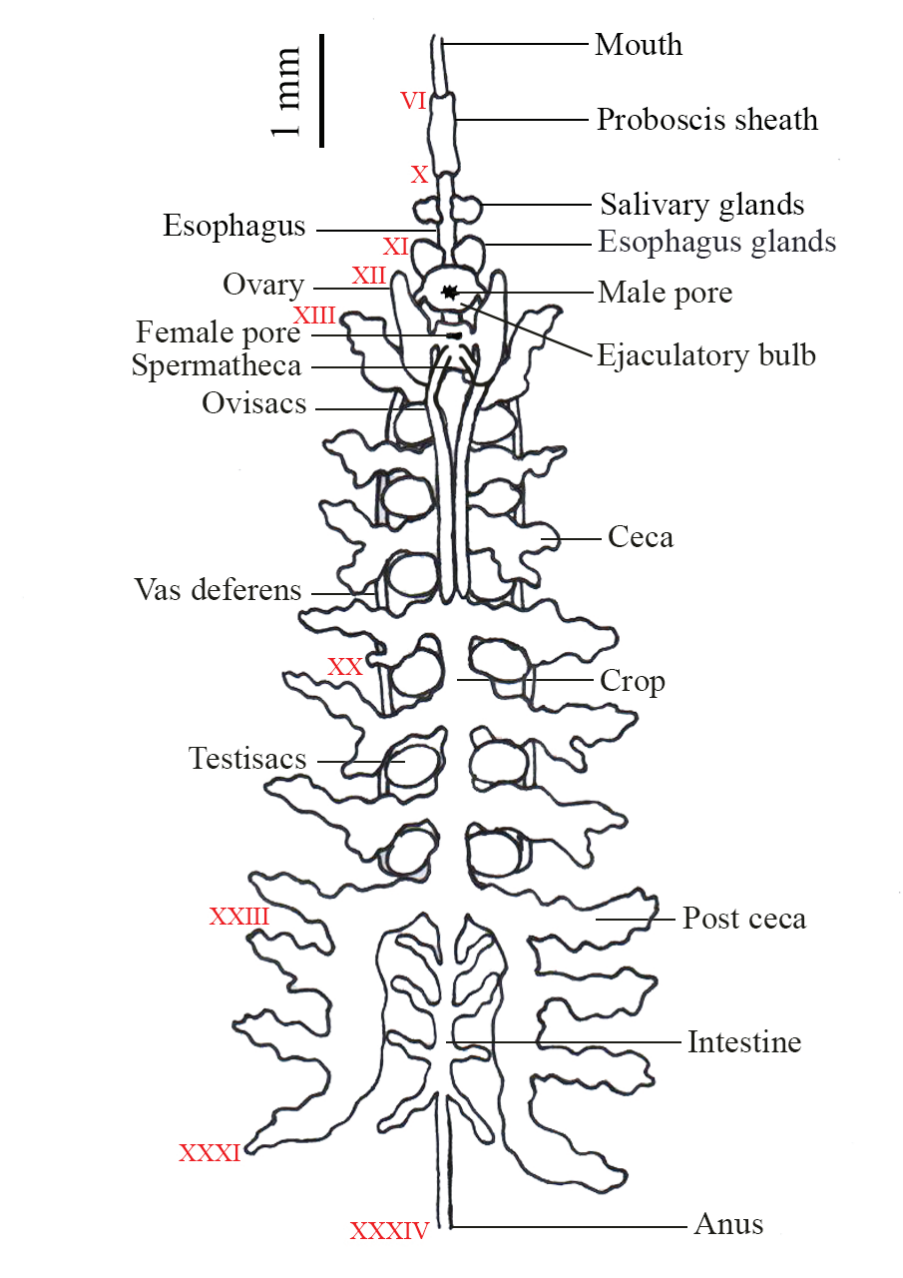
Internal anatomy of *Placobdelloides sirikanchanae* sp. nov.

**Figure 10.**
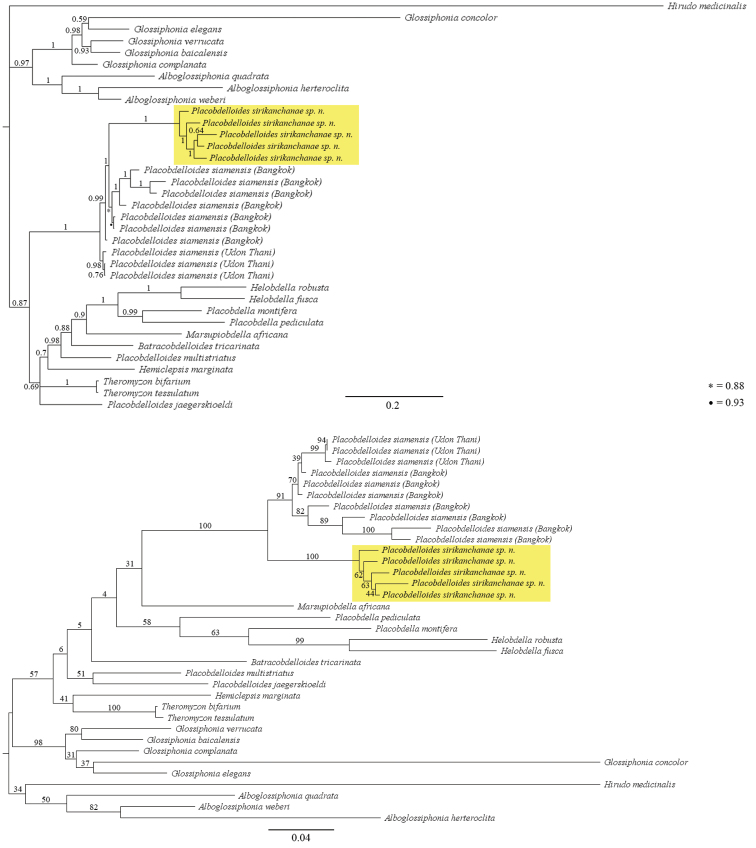
Phylogenetic analysis of the COI-ND1 genes of glossiphoniid leeches. The upper diagram is from the Bayesian analysis; the lower is from the maximum likelihood analysis.

**Table 2.** *P*-distance values of COI genes within (diagonal) and among 4 species of *Placobdelloides* including *P. sirikanchanae* sp. nov. identified in this study.

Species	1	2	3	4
**1***Placobdelloides sirikanchanae* sp. nov.	2.5–6.2%			
**2** *Placobdelloides siamensis*	10.4–27.7%	0.0–10.1%		
**3** *Placobdelloides multistriatus*	19.3–21.7%	15.6–30.6%	–	
**4** *Placobdelloides jaegerskioeldi*	21.0–23.5%	17.3–31.6%	12.6%	–

**Table 3.** *P*-distance values of ND1 genes within (diagonal) and among 4 species of *Placobdelloides* including *P. sirikanchanae* sp. nov. identified in this study.

Species	1	2	3	4
**1***Placobdelloides sirikanchanae* sp. nov.	1.3–3.3%			
**2** *Placobdelloides siamensis*	5.4–6.9%	0.0–1.7%		
**3** *Placobdelloides multistriatus*	15.1–15.8%	15.1–15.4%	–	
**4** *Placobdelloides jaegerskioeldi*	15.1–16.0%	13.4–13.6%	14.3%	–

##### Type host.

Dark-bellied leaf turtles (*Cyclemys enigmatica*).

##### Additional host.

Asian leaf turtles (*C. dentata*).

##### Habitat.

*Placobdelloides sirikanchanae* sp. nov. can be found attached on the shell surface, both the carapace and plastron, of *C. dentata* and *C. enigmatica*, which inhabit the bottom of enclosed shallow muddy puddles on rubber plantations. In the rainy season, several puddles will be connected due to an increase in the water level. Numerous small vertebrates are present in these puddles, such as small fishes or tadpoles. In the dry season, the puddles will be disconnected as the shallower waters disappear from evaporation. These aquatic ecosystems usually have low oxygen due to decomposition of leaf litter and nonflowing water.

##### Laboratory observations.

Ten individuals of *P. sirikanchanae* sp. nov. were released into a tank with water from the type locality and equipped with an oxygen pump. All ten died almost immediately. The ten remaining specimens survived in a sealed bottle under low dissolved oxygen conditions. No ventilation (undulating movement display) was observed. After three days, they initiated copulation and deposited eggs in the sealed bottles.

##### Reproduction.

**Figure 11.**
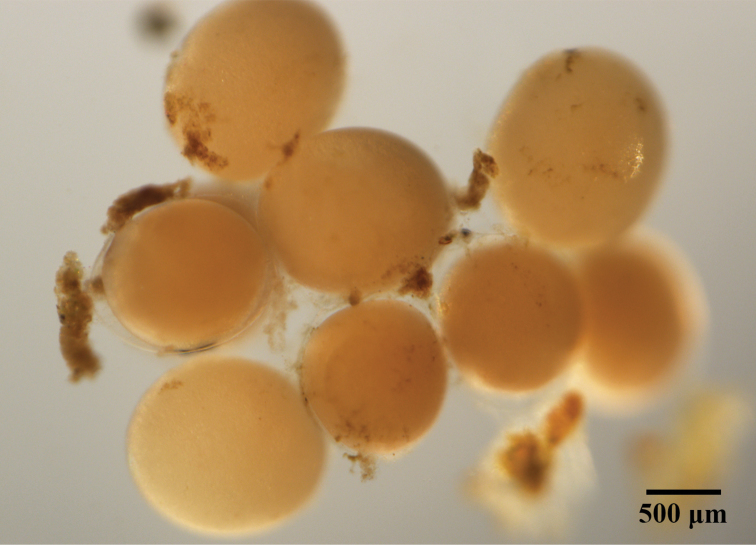
Two-day old creamy whitish coloured eggs of *Placobdelloides sirikanchanae* sp. nov. after deposition.

Ten individuals of *P. sirikanchanae* sp. nov. displayed reproductive activity in a sealed bottle (low oxygen condition). One copulated with another individual for a few hours before they separated. The beginning of gestation was observed inside the ovisacs of both individuals (seen through the ventral surface) 2–3 days after copulation and gestation continued for approximately 3–4 days more before deposition of eggs. Round creamy-colored eggs, approximately 104–115 eggs per individual, were deposited and aggregated inside the transparent membrane beneath the venter groove of the parent (Figure 11). Eggs were incubated for 3–4 days before hatching. Juveniles remained beneath the ventral groove of the parent for 10–15 additional days before leaving the parent and living on their own.

##### Etymology.

The species is named in honor of Associate Professor Prapaisiri Sirikanchana, the pioneer aquatic parasitologist of Thailand. The following common names, *Sirikanchana’s leech* (English), *Pling Arjan Prapaisiri* (Thai), and *Sirikanchanas Plattegel* (German) are suggested.

##### Remarks.

*Placobdelloides sirikanchanae* sp. nov. was distinguished from *P. siamensis* (based on the original description by Oka (1917) and the re-description by Chiangkul et al. (2018)) based on the following combination of characteristics (Table 4): elongated narrow body, smooth anterior sucker surface with numerous pits inside, 13–17 well-developed knob papillae on each annulus, 69 total annuli, dark brown to greenish color when live with a crimson median line, male gonopore between XIa1/a2 (annuli 23 and 24), female gonopore between XIa3/XIIa1 (annuli 25–26), anus opening between the last annulus and the posterior sucker, rough posterior sucker surface with random pits, and 104–115 eggs per clutch. In addition, *P. sirikanchanae* was found on *C. dentata* and *C. enigmatica*, which inhabit the bottom of enclosed shallow muddy puddles on rubber plantations, differing from *P. siamensis*, in that it is found on *Cuora amboinensis*, *Heosemys annandalii*, *Malayemys macrocephala*, *M. subtrijuga*, *M. khoratensis*, and *Siebenrockiella crassicollis* inhabiting larger, more open ponds.

**Table 4.** Comparison of morphological characters, egg number per clutch, host, and distribution of *Placobdelloides sirikanchanae* sp. nov. and *P. siamensis* (Oka, 1917) in Thailand.

Characters	*P. sirikanchanae* sp. nov.	*P. siamensis* (Oka, 1917)	
		Oka 1917	Chiangkul et al. 2018
Host	*Cyclemys dentata* and *C. enigmatica*	*Siebenrockiella crassicollis*	*Cuora amboinensis*, *Heosemys annandalii*, *Malayemys macrocephala*, *M. subtrijuga*, *M. khoratensis*, and *S. crassicollis*
Distribution	Sadao, Songkhla	Lampam, Pattalung	Bangkok and Udon Thani
Maximum relaxed length (mm)	40.39	15.00	25.00
Maximum relaxed widest width (mm)	4.89	4.00	5.57
Body shape	Elongated narrow	Elongated oval	Elongated oval
Eye location	III	III	III
Anterior sucker diameter (mm)	1.08	2.50	1.86
Anterior sucker surface	Smooth with numerous pits	–	Smooth with numerous pit
Position of proboscis opening	Posterior subterminal	Posterior subterminal	Posterior subterminal
Number of dorsal papillae on each annulus	13–17	22–27	5–9
Shape of dorsal papillae	Well-developed knob shape	Cone shape	Well-developed longitudinal rod shape
Total annuli	69	67	69
Live dorsal color	Dark brown greenish with crimson median line	Uniform gray with faint brown median line (in alcohol)	Brownish gray with yellow median line and four pairs of scarlet dots
Male gonopore location	XIa1/a2 (annuli 23/24)	XIa3/XIIa1 (annuli 25/26)	Xa3/XIa1 (annuli 22/23)
Ejaculatory bulb	Apple-like sac	–	Glasses-like sac
Female gonopore location	XIa3/XIIa1 (annuli 25/26)	XIIa2/a3 (annuli 27/28)	XIa2/a3 (annuli 24/25)
Spermatheca	Rectangular sac	–	Slender sac
Anus location	Between last annuli and posterior sucker	Between last annuli and posterior sucker	Between last annuli and posterior sucker
Posterior sucker diameter (mm)	1.94	3.00	3.00
Posterior sucker surface	Rough with random scattered pits	–	Smooth with random scattered pits
Eggs per clutch	104–115	–	173–412

## Discussion

**Figure 12.**
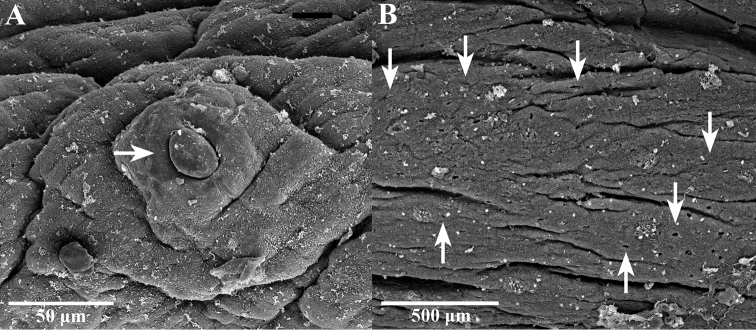
Scanning electron micrograph of *Placobdelloides siamensis* from previous study (Chiangkul et al. 2018) **A** dorsal papillae (arrow) **B** smooth surface with randomly scattered pits (arrows).

*Placobdelloides sirikanchanae* sp. nov. was identified as a new leech species based on morphological and genetic characteristics and was shown to be distinct from other members of its genus. Comparison of *P. sirikanchanae* with other species of *Placobdelloides* that parasitize crocodiles and turtles revealed the following: *P. bancrofti* is distinguished from *P. sirikanchanae* by having one annulus separating the male and female gonopores and an absence of dorsal papillae; *P. emydae* has a slightly dilated head and three pairs of metameric papillae on the dorsum; *P. fimbriata* has a unique gill-like marginal fringe; *P. multistriata* has two pairs of salivary glands and the absence of dorsal papillae; the original description of *P. siamensis* from the description by Oka (1917) has an elongated oval shape, 22–27 cone papillae, and a different gonopore distribution; *P. siamensis* based on the description by Chiangkul et al. (2018), has an elongated oval shape, yellow median line, numerous scattered yellow pigments on dorsal, 5–9 well-developed rod papillae, a different gonopore distribution, and smooth posterior sucker with random pits (Figures 8, 12, clarified from previous study); and *P. stellapapillosa*Govedich et al. 2002 has a proboscis opening on the anterior subterminal mouth and unique star-shaped papillae (Oka 1917; Harding and Moore 1927; Best 1931; Sawyer 1986; Govedich et al. 2002; McKenna et al. 2005).

The phylogenies (Fig. 10) obtained in this study revealed the monophyletic relationship of *Placobdelloides* species that inhabit Thailand. The phylogenetic trees clearly indicated the divergence between *P. sirikanchanae* and *P. siamensis* (Bangkok and Udon Thani population) by having a high percentage of differences between the species for both the COI and ND1 gene. However, after several attempts, we were unable to retrieve the topotype of *P. siamensis* from Pattalung and could not conduct the sequence comparisons, but the morphological characters of *P. siamensis* from the other localities are clear and easily differentiate it from *P. sirikanchanae*. According to the phylogenetic analysis, *P. sirikanchanae* is the sister taxon of *P. siamensis* (Bangkok population).

This is the first report of the reproductive biology of *P. sirikanchanae*. This hermaphroditic leech displayed monandrous copulation and exchanged pseudospermatophores with other leeches a few hours before separation . The gestational period after copulation through egg deposition was approximately 5–7 days, which began in the ovisacs beginning 2–3 days after copulation. The family Glossiphoniidae is unique in that members of this family exhibit parental care of their eggs and juveniles (Sawyer 1971). Compared to other glossiphoniid leeches, *P. sirikanchanae* had more eggs per clutch (104–115 eggs per clutch) than *Glossiphonia complanata* (60 eggs per clutch) or *Helobdella stagnalis* (Linnaeus, 1758) (50 eggs per clutch) but fewer than *P. stellapapillosa* (100–200 eggs per clutch) and *P. siamensis* (173–412 eggs per clutch) (Kutschera and Wirtz 2001; Chiangkul et al. 2018). For the incubation period, *P. sirikanchanae* had a shorter period from egg deposition through juvenile hatching (3–4 days) compared to *H. robusta* (9 days and 13 hr) (Weisblat and Huang 2001). For the parental care period, it had a shorter period from egg deposition through separation of juveniles from the parent (13–19 days) than *G. complanata* (30 days) and *H. stagnalis* (45–50 days) (Sawyer 1986; Kutschera 1992). Therefore, *P. sirikanchanae* might currently have the smallest number of eggs per clutch in the genus *Placobdelloides* and the shortest periods of incubation and parental care in the family Glossiphoniidae.

This is the first report of *P. sirikanchanae* parasitizing Asian leaf turtles (*C. dentata*) and dark-bellied leaf turtles (*C. enigmatica*). In the field surveys of this study, both the leech and the turtles inhabited the bottom of enclosed shallow muddy puddles or patches in rubber plantations. Small puddles and patches are a temporary aquatic system that usually occurs after rain and disappears within a few weeks or months from evaporation or seeping into the ground. In addition, this aquatic system usually has low dissolved oxygen conditions from leaf decomposition and the absence of flowing water, but despite this, there were numerous small vertebrates living there, such as fishes and *Rhacophorus* tadpoles (Shahriza et al. 2010).

For *P. sirikanchanae*, its small clutch size and faster development times might be an adaptation to living in these temporary ponds. Moreover, the observed behavior in the laboratory combined with water conditions in the field indicated that *P. sirikanchanae* is a leech that can tolerate low dissolved oxygen conditions.

*Cyclemys dentata* and *C. enigmatica* are members of the family Geoemydidae, the main freshwater turtle family found in Thailand, along with *Cuora amboinensis*, *Heosemys annandalii*, *Malayemys macrocephala*, *M. subtrijuga*, *M. khoratensis*, and *Siebenrockiella crassicollis*, all of which are the hosts of *P. siamensis* (Oka 1917; Chiangkul et al. 2018). However, most host turtles of *P. siamensis* usually inhabit ponds, lakes, or rivers that have flowing water and differ from the habitats of *C. dentata* and *C. enigmatica* (Das 2010; Fritz et al. 2008). Accordingly, the habitat preferences of host turtles also support the identification of *Placobdelloides* leech parasites in Thailand.

## Supplementary Material

XML Treatment for Placobdelloides

XML Treatment for Placobdelloides sirikanchanae
